# Comparative Immunopathology of *Toxoplasma gondii* Natural Infections in Marmosets (*Callithrix* spp.) and Howler Monkeys (*Alouatta* spp.), Brazil

**DOI:** 10.1111/jmp.70112

**Published:** 2026-09-04

**Authors:** Marina Pellegrino, Alessandra L. M. Santos, Cinthya dos Santos Cirqueira, Gabrielle F. P. da Silva Gagliotti, Ana Carolina Ewbank, Carlos Sacristán, Rodrigo Albergaria Ressio, Natália C. C. A. Fernandes, Juliana Mariotti Guerra, José Luiz Catão‐Dias

**Affiliations:** ^1^ Laboratory of Wildlife Comparative Pathology, School of Veterinary Medicine and Animal Sciences University of São Paulo São Paulo Brazil; ^2^ Pathology Center Adolfo Lutz Institute São Paulo Brazil; ^3^ Group of Epidemiology and Environmental Health Animal Health Research Center (INIA‐CISA/CSIC) Madrid Spain

**Keywords:** immunohistochemistry, immunology, neotropical primates, protozoa, toxoplasmosis

## Abstract

**Introduction:**

Neotropical primates (NTPs) present varying susceptibility to toxoplasmosis caused by *Toxoplasma gondii*, but their immunopathogenic responses remain poorly understood.

**Materials and Methods:**

Local immune responses were characterized in the liver, spleen, and central nervous system (CNS) of 50 naturally infected NTPs (31 *Callithrix* spp.; 19 *Alouatta* spp.) by immunohistochemistry. Splenic white pulp morphometry, CD3+ T lymphocyte, Iba1+ macrophage/microglia, polymorphonuclear neutrophil quantification, and IFN‐γ and IL‐10 semi‐quantification were performed.

**Results:**

Both genera exhibited splenic white pulp hyperplasia. Within genera, marmosets showed predominant CD3+ infiltrates in the spleen and histiocytic infiltrates in the liver and CNS, whereas howler monkeys exhibited predominantly histiocytic infiltrates in all tissues. Between genera, marmosets had significantly higher splenic CD3+ T‐cell (*p* < 0.05), Iba1+ macrophage (*p* = 0.030), and IFN‐γ (*p* = 0.018) immunolabeling than howler monkeys.

## 1 Introduction

Toxoplasmosis is a zoonotic disease caused by *Toxoplasma gondii* (*T. gondii*) (phylum Apicomplexa), an obligate intracellular parasite with felids as definitive hosts [1]. Pathogenesis and disease outcome are influenced by host‐specific factors and parasite genetic diversity [2]. Whereas most domestic animals are resistant, certain wildlife groups—including Australian marsupials, Madagascan lemurs, and neotropical primates (NTPs)—are highly susceptible [3, 4, 5].

NTPs represent approximately 30.5% of all non‐human primate (NHP) taxa and are distributed across Mexico, Central America, and South America, with Brazil harboring 25% of the world's species [3]. Infectious diseases, including toxoplasmosis, are important threats to NTPs conservation, with outbreaks documented in both captive and free‐living individuals across species with varying outcomes [6, 7, 8, 9, 10].

Marmosets (family Callitrichidae, genus *Callithrix*) are highly susceptible to toxoplasmosis and frequently develop acute, fatal disease, whereas howler monkeys (family Atelidae, genus *Alouatta*) present variable outcomes [3, 6, 7]. These differences in susceptibility and clinical response may be influenced by evolutionary history, host genetics, and ecological adaptations [3]. In other species, hosts' immune status and responses have also been linked to toxoplasmosis susceptibility, with the balance between Th1 and Th2 cytokines playing a central role in disease outcome [11, 12, 13].

The immune response against *T. gondii* is intense and cell‐mediated, with T lymphocytes playing a central role in parasite control. During the early stages of disease, the parasite is recognized by innate immune cells that initiate the production of IL‐12, which induces the production of IFN‐γ, a pro‐inflammatory Th1 cytokine, whose role is to inhibit the parasite replication, but when unregulated, it can drive severe immunopathology [14]. The acute phase of disease is characterized by high levels of IFN‐γ, IL‐12, TNF‐α, IL‐6, and IL‐1 pro‐inflammatory cytokines and the IL‐10 anti‐inflammatory cytokine. IL‐10 is a regulatory cytokine that limits severe immunopathology by modulating the Th1 response [11]. The balance between these cytokines is essential to control parasite replication without causing tissue damage. Characterizing these cytokines and cell populations in target organs may provide important insights into the mechanisms underlying disease severity in these NTP genera.

The parasitic forms of *T. gondii* (i.e., tachyzoites and bradyzoites) can disseminate to virtually any organ [15]. In marmosets, the liver is a primary site of acute replication and *T. gondii* forms are also frequently detected in the spleen [7, 8]. Furthermore, the central nervous system (CNS) is also a parasite target, with severe lesions described in brains of infected NTPs [7, 8, 16, 17]. Given that these three organs are targets of *T. gondii* forms, they constitute relevant sites for characterizing the local immune response underlying disease severity. Although pathological lesions caused by *T. gondii* in NTPs are well documented [6, 7, 15, 16, 17, 18], no study has directly compared immunophenotypic profiles between NTPs susceptible genera or characterized tissue‐specific immune responses within a single genus.

Thus, we hypothesized that marmosets and howler monkeys present different local tissue immune responses to toxoplasmosis. We quantitatively assessed CD3+ (T lymphocytes), Iba1+ (macrophage/microglia), and polymorphonuclear neutrophil (PMNs) cell densities in liver, spleen, and CNS, performed splenic white pulp morphometry, and semi‐quantified IFN‐γ and IL‐10 expression by immunohistochemistry (IHC) in the spleen, in naturally infected individuals of both genera. We aimed to quantitatively characterize and compare the tissue‐specific immune profile associated with naturally occurring toxoplasmosis within and between two genera of NTP with different susceptibility to the disease.

## 2 Material and Methods

### 2.1 Study Group

Biological samples from naturally 50 *T. gondii*‐infected NTPs (31 marmosets and 19 howler monkeys) with previously confirmed histopathological, IHC, and molecular diagnoses in spleen, liver, or CNS were retrieved from the Adolfo Lutz Institute (IAL) tissue bank (January 2015–June 2020) as part of the Brazilian National Surveillance Program of Epizootics. Animals included both free‐living and captive individuals from the states of Distrito Federal, São Paulo, Santa Catarina, Sergipe, and Rio Grande do Sul. Inclusion criteria comprised previous absence of histopathological diagnosis for co‐morbidities, availability of hematoxylin‐eosin (HE) slides with at least one target tissue, and sufficient formalin‐fixed paraffin‐embedded (FFPE) material for IHC. Animals showing moderate‐to‐advanced tissue autolysis were excluded.

A total of 43 *T. gondii*‐uninfected NTPs (29 marmosets and 14 howler monkeys) from the same biobank, showing absence of splenic alterations and negative histopathological findings for *T. gondii* and other relevant infectious agents, were included exclusively as controls for splenic white pulp area comparisons. The control group formed by uninfected animals was matched to the infected group by genus and year of death to minimize temporal and taxonomic bias.

Biological information such as species/genus identification, sex, age class, and origin was retrieved from submission forms (SINAN—Sistema Nacional de Agravos de Notificação) (see Tables S1 and S2).

### 2.2 Histopathology and Splenic White Pulp Morphometry

Splenic white pulp follicle (SWPF) area was measured in the *T. gondii*‐uninfected NTPs that served as controls and in the 46 NTPs (18 howler monkeys; 28 marmosets) *T. gondii*‐infected. Four cases out of the total were excluded due to the absence of HE slides with splenic tissues. Five fields per slide were photographed at 10× magnification (OPTICAM software, ×64, v4.10.17015). Follicle major and minor axes were measured and SWPF area calculated as *A* = π × *r* ^2^. The mean SWPF area per animal was compared between uninfected and infected animals of each genus.

### 2.3 Immunohistochemistry

Tissue sections (4 μm) were deparaffinized, rehydrated through graduated alcohols, and submitted to antigen retrieval in a pressure cooker with citric acid solution (pH 6.0). Endogenous peroxidase was blocked with 6% H_2_O_2_ in two 20‐min baths (first at 37°C, second at room temperature). Primary antibodies (Table 1) were applied and incubated overnight (18 h, 4°C). Amplification and visualization used the Complement Reveal reagent and Reveal HRP Conjugate (DCMT‐999 and DHRR‐999, Spring Bioscience) followed by 3,3′‐diaminobenzidine tetrahydrochloride (DABC‐004 and DABS‐999, Spring Bioscience). Slides were counterstained with Harris' Hematoxylin, dehydrated, and mounted with Entellan resin (Merck, Germany).

**TABLE 1:** Technical specifications of primary antibodies.

Antibody	Species	Type, clone or code	Dilution
Anti‐CD3^a^	Mouse	Pab, A0452	1:300
Anti‐Iba1^b^	Rabbit	Pab, A1239	1:200
Anti‐IL10^c^	Mouse	Mab, QS‐10	1:50
Anti‐IFN γ^c^	Mouse	Mab, MD‐1	1:50

Abbreviations: CD, cluster of differentiation; Iba, ionized calcium‐binding adaptor; IFN, interferon; IL, interleukin; Mab, monoclonal antibody; Pab, polyclonal antibody.

^a^ Dako‐Agilent (Santa Clara, CA, USA).

^b^ AbClonal Technology (Woburn, MA, USA).

^c^ U‐CyTech biosciences (Utrecht, the Netherlands).

The same IHC protocol was applied across all tissues of infected animals, with positive and negative controls included in each staining run. Immunolabeling evaluation included semi‐quantitative cytokine scoring and immune cell counting according to predefined criteria.

### 2.4 Inflammatory Cells Quantification

Ten randomly selected fields per tissue were photographed at 400× magnification using OPTICAM software for cell quantification. PMNs were identified in HE‐stained slides based on morphological criteria and photographed for quantification in 5 fields of red pulp and 5 fields of white pulp in the spleen, 10 fields of hepatic necrotic areas, and 10 fields of inflammatory infiltrates in the CNS. Immunopositive CD3+ T lymphocytes and Iba1+ microglia/macrophages were quantified in 10 randomly selected fields of the spleen (red pulp and white pulp), liver (centrilobular, periportal, and necrotic areas), and CNS by immunolabeling in IHC slides. Cell counts were performed using QuPath v0.5.1 [19]. The per‐animal mean cell counts across the 10 selected fields for each tissue served as the representative value for subsequent analyses.

### 2.5 Semi‐Quantitative Cytokine Assessment

Cytokine immunolabeling was evaluated exclusively in naturally infected animals to characterize the local cytokine response during active toxoplasmosis. One *T. gondii*‐negative individual per genus was included solely as a qualitative reference to confirm staining specificity and pattern and was not used for statistical comparison. IFN‐γ and IL‐10 immunolabeling were initially evaluated in liver, spleen, and CNS tissues. However, only splenic samples showed consistent immunoreactivity suitable for semi‐quantitative analysis. Therefore, cytokine scores were restricted to splenic tissue. Scores were calculated by multiplying the percentage of positive cells (0: none; 1: < 10%; 2: 11%–50%; 3: 51%–80%; 4: > 81%) by staining intensity (0: none; 1: weak; 2: moderate; 3: strong), as described by Guerra et al. [20] Score standardization was validated by an experienced pathologist.

### 2.6 Statistical Analysis

Normality was assessed independently for each comparison using the Shapiro–Wilk test. SWPF area was evaluated using the Mann–Whitney *U*‐test, except for the comparison between infected and control howler monkeys, which met the assumption of normality and was therefore analyzed using Welch's *t*‐test. Intergeneric inflammatory cell comparisons were performed with the Mann–Whitney *U*‐test or Student's *t*‐test, as appropriate. The Kruskal–Wallis test was used to compare cell type distributions within tissues of each genus. Cytokine score differences between groups were evaluated with the Mann–Whitney *U*‐test. Spearman's rank correlation was used to assess associations between cytokine scores and cell counts. Results of parametric tests were reported as mean and standard deviation, while non‐parametric tests were reported as median and interquartile range (IQR). Statistical significance was set at *p* < 0.05. All analyses were performed in R (v4.4.1).

## 3 Results

### 3.1 Splenic White Pulp Morphometry

**FIGURE 1:**
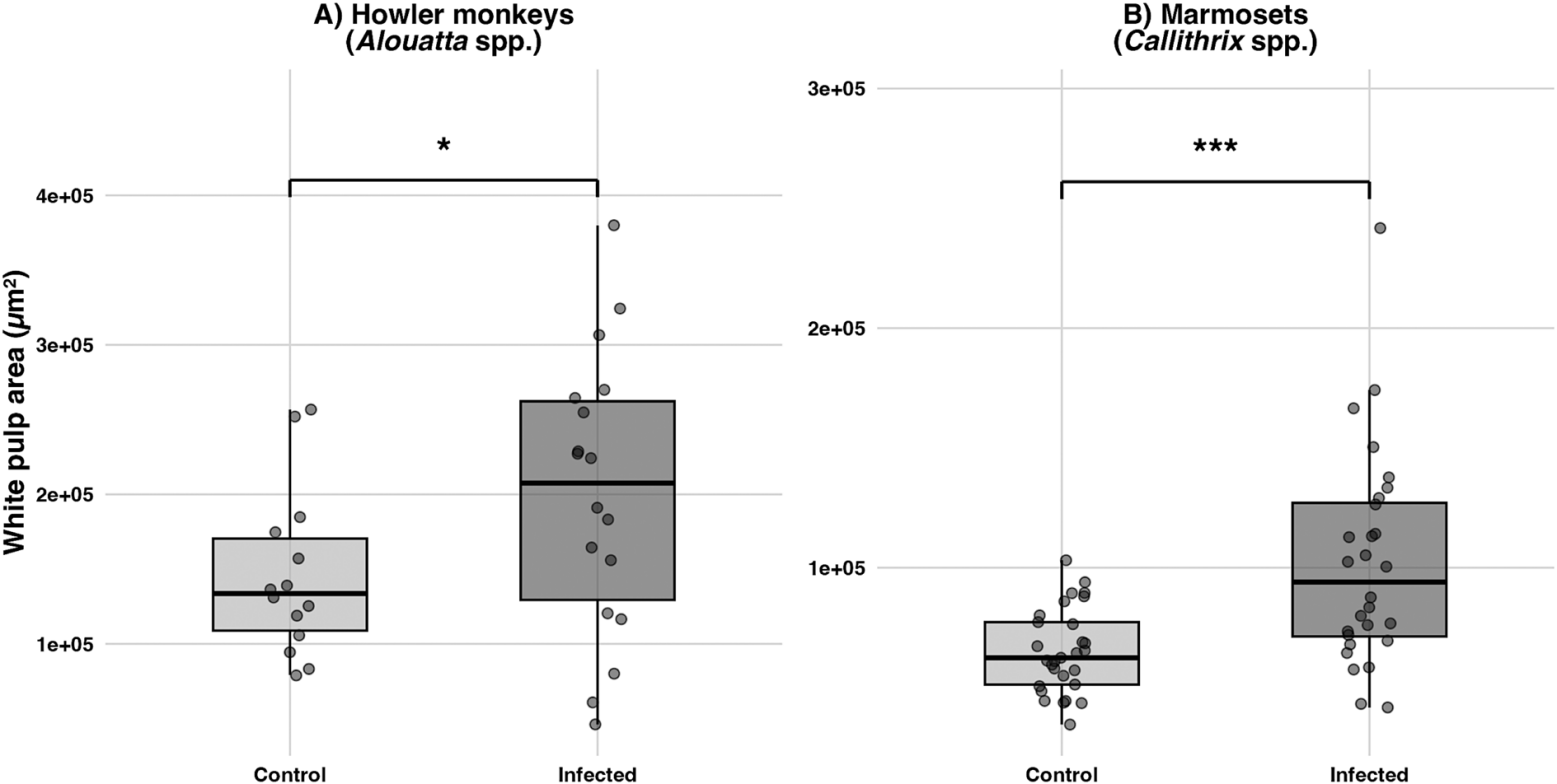
Comparison of the splenic white pulp area (μm^2^) between control and naturally *Toxoplasma gondii*‐infected (A) howler monkeys (*Alouatta* spp.) and (B) marmosets (*Callithrix* spp.). Each dot represents one animal. Statistical comparisons were performed using Welch's *t*‐test for howler monkeys and the Mann–Whitney *U*‐test for marmosets. *p < 0.05; ***p < 0.001.

In the control group, howler monkeys (median: 133 659.85, IQR [108 864.63–170 438.35]) presented significantly larger splenic white pulp follicle areas (μm^2^) than marmosets (median: 62 403.8, IQR [51 231.1–77 276.6]) (*p* < 0.001). When systemic toxoplasmosis was present, howler monkeys (mean ± SD: 200 072.07 ± 93 212.15) presented significantly larger white pulp follicle areas compared to uninfected howler monkeys (145 619.41 ± 55 634.88) (*p* = 0.049) (Figure 1A). Similarly, marmosets with systemic toxoplasmosis (median: 94 032.64; IQR [71 331.81–127 067.08]) presented significantly larger white pulp follicle areas compared to their respective controls (*p* < 0.001) (Figure 1B). Marked necrotizing splenitis with loss of normal splenic architecture was observed in some infected animals.

### 3.2 Inflammatory Cell Distribution According to Tissue and Genera

**FIGURE 2:**
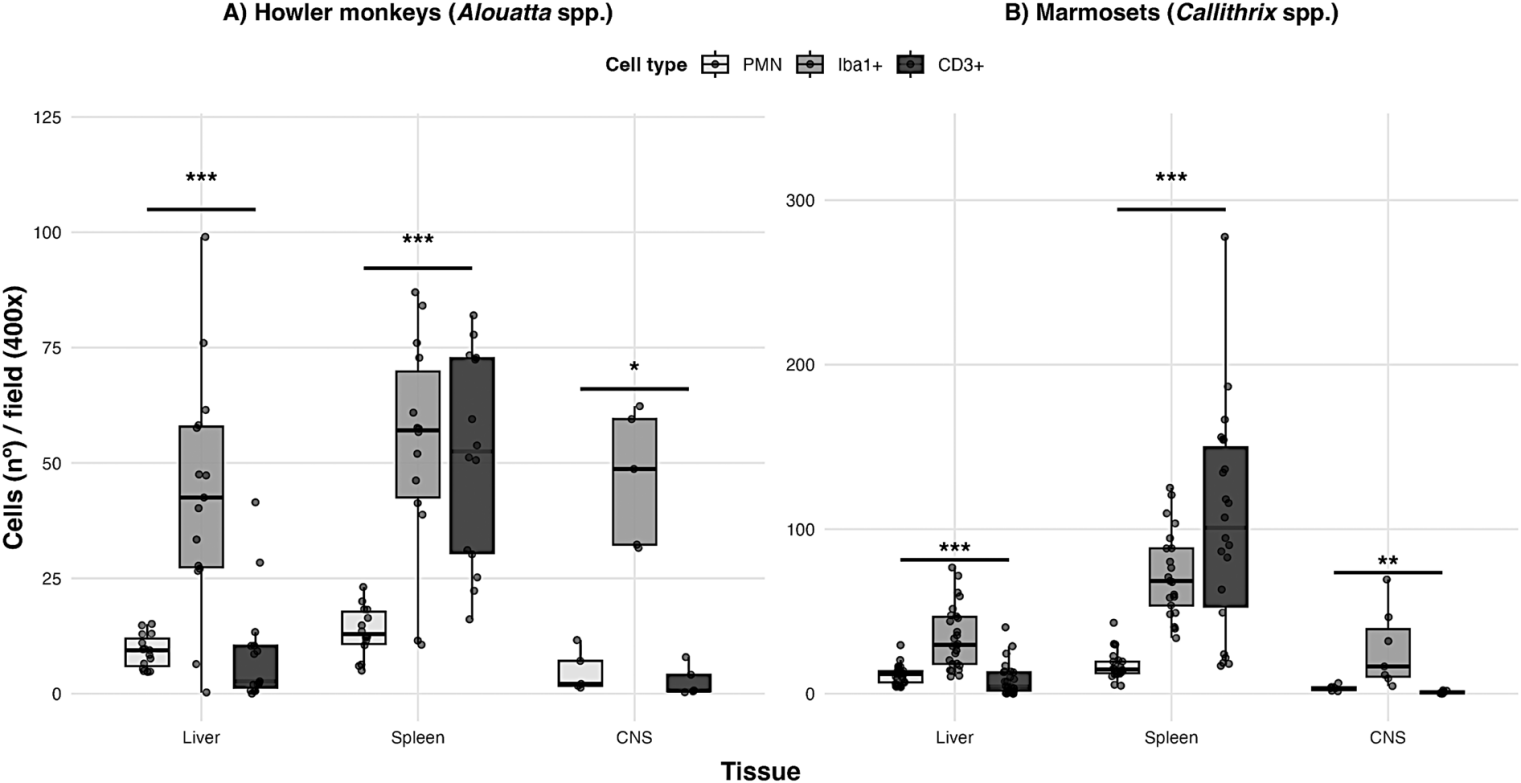
Comparison of polymorphonuclear neutrophil (PMN), Iba1+ and CD3+ cell counts within the liver, spleen, and central nervous system (CNS) of naturally *Toxoplasma gondii*‐infected (A) howler monkeys (*Alouatta* spp., *n* = 18) and (B) marmosets (*Callithrix* spp., *n* = 29). Points represent individual field values. Cell counts are expressed as number of cells per field (400× magnification). Statistically significant difference among the three cell types within each tissue by the Kruskal‐Wallis test (*p < 0.05, **p < 0.01, ***p < 0.001).

Iba1+ macrophages/microglia were significantly more abundant than CD3+ T lymphocytes and PMNs in the spleen (*p* < 0.001), the liver (*p* < 0.001), and the CNS (*p* = 0.009) of howler monkeys. In marmosets, CD3+ T lymphocytes predominated over the two cell types in the spleen (*p* < 0.001), while Iba1+ macrophages/microglia were more abundant in the liver (*p* < 0.001) and in the CNS (*p* < 0.001) (Figure 2). CD3+ T lymphocytes were mostly present in the splenic white pulp, and Iba1+ cells were particularly abundant in hepatic periportal zones, splenic red pulp inflammatory infiltrates, and CNS neuroparenchyma in both genera (see Figures S1 and S2). Complete statistical results are provided in Table S3.

### 3.3 Inflammatory Cell Type Comparison Between Genera

**FIGURE 3:**
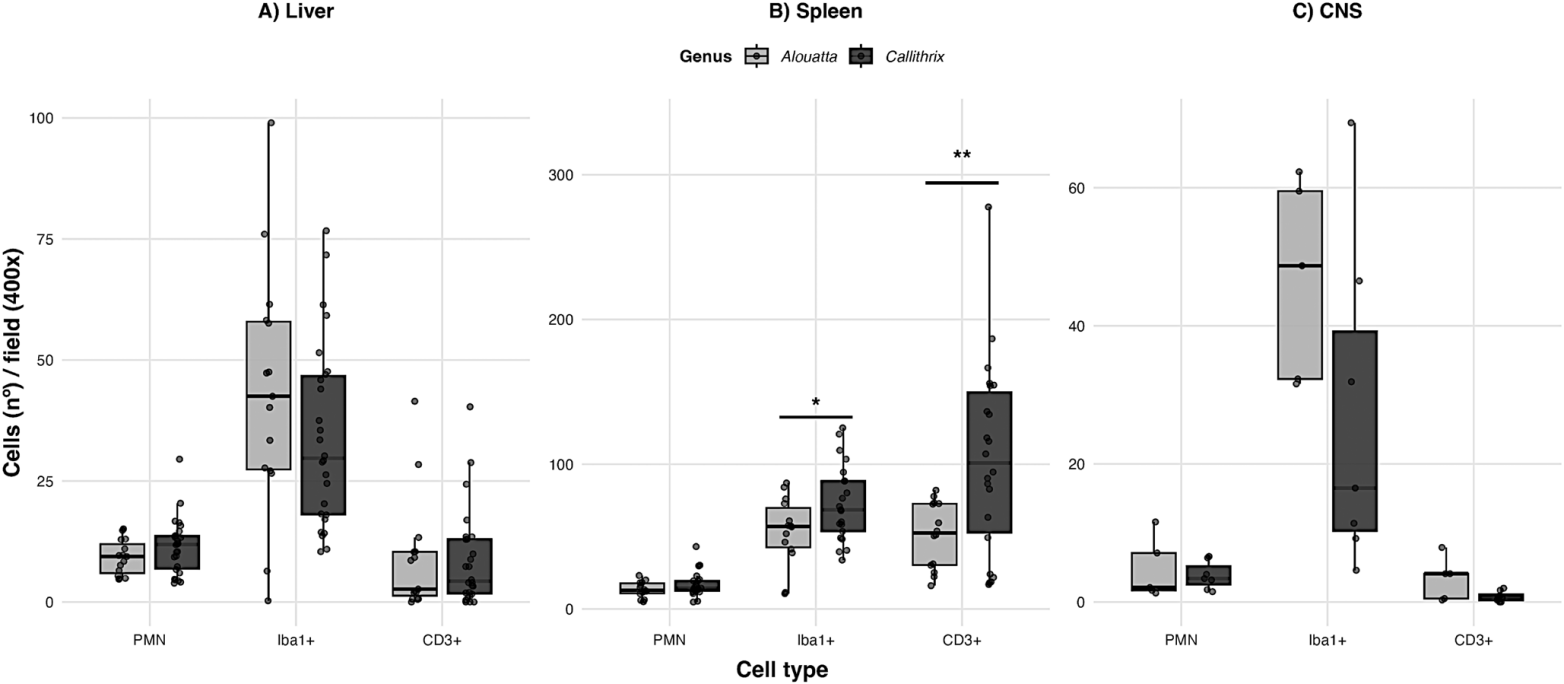
Comparison of polymorphonuclear neutrophil (PMN), Iba1+, and CD3+ cell counts between naturally *Toxoplasma gondii*‐infected howler monkeys (*Alouatta* spp.) and marmosets (*Callithrix* spp.). Cell counts were evaluated in the (A) liver, (B) the spleen, and (C) the central nervous system (CNS) and are expressed as the number of cells per 400× field. Statistically significant differences between genera for each cell type by the Student's *t*‐test (*p < 0.05; **p < 0.01).

PMNs were distributed mainly in hepatic necrotic areas and splenic inflammatory infiltrates, with rare polymorphic infiltrates in the CNS in both genera, and no significant difference detected in any tissue (*p* > 0.05). Marmosets had significantly higher quantities of CD3+ T lymphocytes (*p* = 0.002) and Iba1+ macrophages (*p* = 0.002) in the spleen than howler monkeys (Figure 3) (see Table S4). Although Iba1+ cell density did not differ significantly between genera in the CNS, these cells were frequently associated with organized nodular gliosis in howler monkeys, whereas marmosets showed diffuse microglial proliferation without structured clustering around lesions.

### 3.4 Semi‐Quantitative Cytokine Expression

**FIGURE 4:**
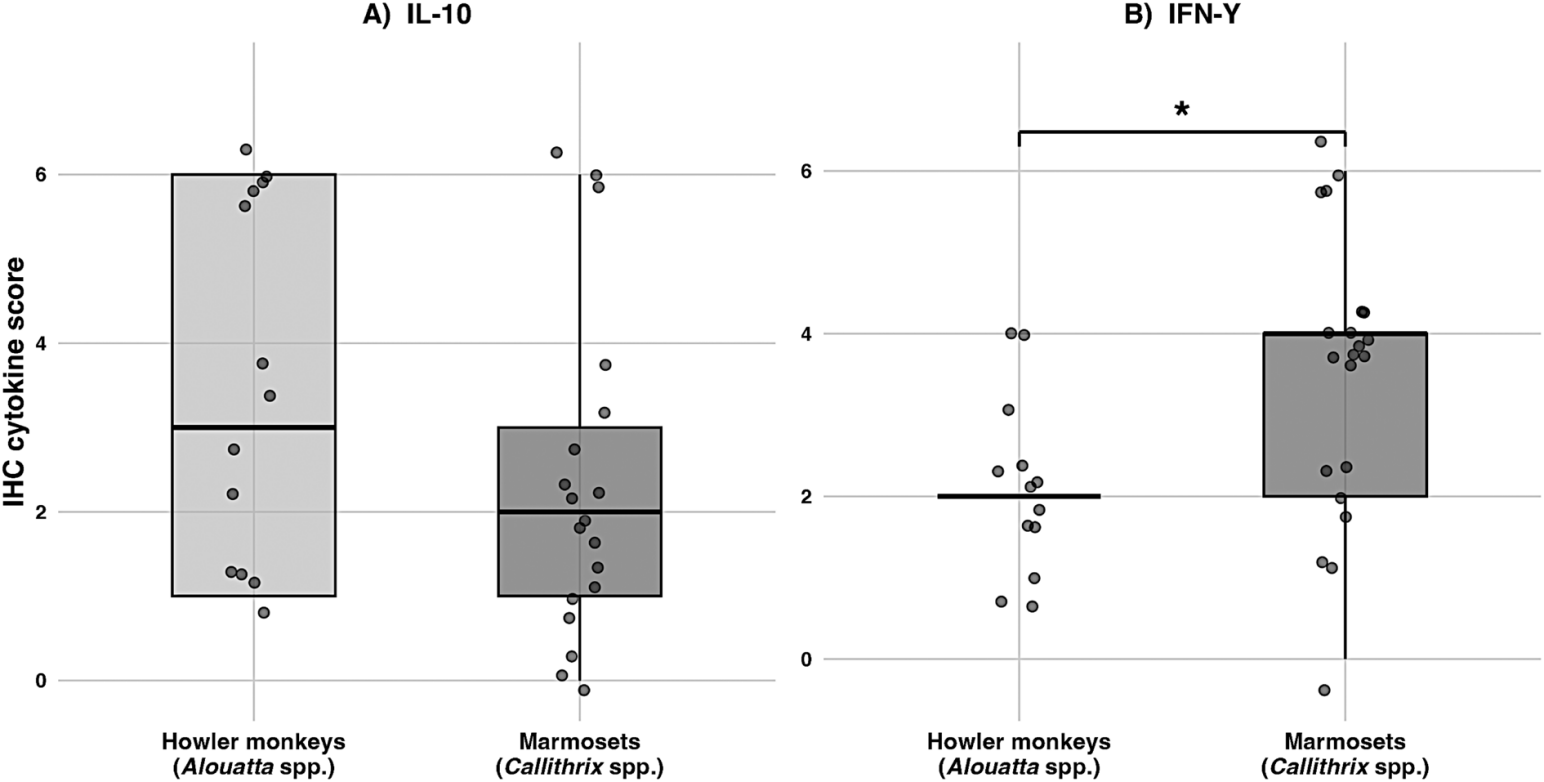
Comparison of (A) IL‐10 and (B) IFN‐γ immunohistochemical (IHC) cytokine score between naturally *Toxoplasma gondii*‐infected howler monkeys (*Alouatta* spp.) and marmosets (*Callithrix* spp.). Statistically significant differences between genera by the Mann–Whitney *U*‐test (*p < 0.05).

Overall, splenic IL‐10 and IFN‐γ IHC scores were low in both genera, with most individuals showing mild staining and few immunolabeled cells (Figure S3). IL‐10+ cells were mainly observed within germinal centers of splenic white pulp. IL‐10 scores did not differ between howler monkeys and marmosets (*p* = 0.147). Within howler monkeys, IL‐10 and IFN‐γ immunolabeling scores were not significantly different (IL‐10: median 3, IQR 1–6; IFN‐γ: median 2, IQR 2–2; *p* = 0.178). IFN‐γ immunolabeling was observed across all splenic compartments, with predominance in the red pulp and mild staining intensity. In marmosets, IFN‐γ immunolabeling scores were significantly higher than IL‐10 scores within the same genus (IFN‐γ: median 4, IQR 2–4; IL‐10: median 2, IQR 1–3; *p* = 0.013), and IFN‐γ scores were also higher than those observed in howler monkeys (*p* = 0.018) (Figure 4). No significant correlations were identified between cytokine immunolabeling scores and CD3+, Iba1+, or PMN cell counts in the spleen.

## 4 Discussion

To our knowledge, this is the first study to quantitatively characterize and compare the local immunophenotypic profile in liver, spleen, and CNS of howler monkeys and marmosets naturally infected with *T. gondii*. Both genera exhibited a predominantly histiocytic inflammatory response in liver and CNS, consistent with prior observations in susceptible NTPs [7, 21] and with the immunological profile described in immunosuppressed or highly susceptible hosts [22]. The higher Iba1+ macrophage counts over CD3+ lymphocytes and PMNs in the spleen of howler monkeys resulted in a predominantly histiocytic inflammatory infiltrate, as previously described in other studies [8, 10, 21]. Meanwhile, marmosets presented higher CD3+ T lymphocyte counts in the splenic tissue, probably reflecting an increase in spleen cellularity, a characteristic of acute toxoplasmosis [23].

Splenic white pulp hyperplasia was present in both genera relative to uninfected animals, reflecting reactive lymphoid expansion during acute infection, as previously documented in callitrichids [6, 24] and consistent with the early spleen response to parasitic infections [23, 24, 25]. Necrotizing splenitis observed in severe cases reflects advanced follicular disruption associated with high parasite burden and intense immune activation [22, 26]. As samples were opportunistically collected from free‐living and captive animals, some of unknown origin, sex, or age, selection bias inherent to field‐based sampling cannot be fully excluded. Although such limitations are intrinsic to opportunistic wildlife studies, they were carefully considered during data interpretation, and findings regarding differences in splenic white pulp area should be interpreted with caution.

The higher CD3+ T lymphocytes and Iba1+ macrophages counts in the spleen of marmosets relative to howler monkeys suggest a more intense splenic immune activation at time of death. During acute toxoplasmosis, increased spleen cellularity reflects early leukocyte recruitment [12], which diminishes as disease progresses [25]. The greater immune cell mobilization in marmosets may therefore reflect an earlier, hyperacute disease stage, consistent with a rapid fatal course, as described in previous reports [7, 24]. Conversely, the organized nodular gliosis observed in the CNS of howler monkeys, in contrast to the diffuse microglial proliferation seen in marmosets, suggests a more chronic, organized local response, which may reflect a longer disease course that allows structured glial reorganization around lesions [22, 27], whereas the diffuse pattern in marmosets may be consistent with a more acute, less organized ongoing response at the time of death [7].

The significantly higher IFN‐γ immunolabeling relative to IL‐10 observed in marmosets spleen is noteworthy. The IFN‐γ cytokine is the main effector against *T. gondii* [11, 28] and is essential for restrincting parasite replication [4]. However, sustained IFN‐γ responses in the absence of adequate IL‐10‐mediated regulation can result in excessive inflammation and severe immunopathology, including hematopoietic suppression, anemia, apoptosis, tissue necrosis, and fatal disease in highly susceptible hosts [12, 29]. Although our findings do not demonstrate cytokine kinetics, the predominance of IFN‐γ immunolabeling over IL‐10 in the spleen is consistent with a stronger pro‐inflammatory response and may reflect the hyperacute course of toxoplasmosis previously described in callitrichids.

The more balanced IFN‐γ and IL‐10 cytokines immunolabeling in howler monkeys, combined with a higher but non‐significant trend toward IL‐10 levels, suggests a more regulated local immune response and a more graduated disease course. These findings support the concept that differences in susceptibility to toxoplasmosis among NTPs are more likely associated with differences in immune regulation rather than parasite recognition [30].

Several methodological limitations should be acknowledged. Cytokine expression was assessed by IHC using a validated semi‐quantitative scoring system [20], which does not allow absolute quantification and is subject to variability related to tissue processing and antibody performance. The anti‐IFN‐γ and anti‐IL‐10 antibodies were not originally developed for NTPs; although binding was confirmed, differences in binding affinity across genera cannot be excluded. In addition, the ordinal nature of the scoring system may limit sensitivity to detect subtle differences. Together, these constraints restrict the inference of cytokine magnitude and immune response based solely on IHC, and the findings should therefore be interpreted with caution. Furthermore, only IFN‐γ and IL‐10 were evaluated, whereas other relevant mediators, such as TNF‐α, IL‐6, and TGF‐β, were not assessed, limiting a broader characterization of the immune profile. The retrospective cross‐sectional design, based on samples collected at death, also precludes assessment of temporal immune dynamics. Future studies employing multiplex cytokine quantification in fresh samples from longitudinally monitored animals would provide more comprehensive and precise immunological insights.

In this study, we have systematically evaluated the immunopathological response of howler monkeys and marmosets to *T. gondii* infection, one of the main threats to neotropical primates' survival. The immune response in both genera was characterized as an acute, predominantly histiocytic inflammatory response, with smaller numbers of T lymphocytes and PMNs, accompanied by reactive hyperplasia of the splenic white pulp. Additionally, the greater immunoexpression of IFN‐γ in marmosets in comparison to howler monkeys and to its own expression of IL‐10 is consistent with the hyperacute and dysregulated immune response previously described in marmosets. This study provides novel comparative immunopathological insights into toxoplasmosis in two susceptible NTP genera and to the body of evidence that wildlife pathology and immunology are indispensable disciplines for the study of NTP health.

## Funding

This research was supported by the Fundação de Amparo à Pesquisa do Estado de São Paulo (FAPESP; grant #2022/08313‐7). This study was financed in part by the Coordenação de Aperfeiçoamento de Pessoal de Nivel Superior ‐ Brasil (CAPES) ‐ Financial Code 001. J. L. Catão‐Dias is a Conselho Nacional de Desenvolvimento Científico e Tecnológico (CNPq) research fellow (grant #304106/2022‐4). A. C. Ewbank holds a Juan de la Cierva Formación fellowship (JDC2022‐048632‐I) granted by Agencia Estatal de Investigación, Ministerio de Ciencia, Innovación y Universidades, Spain.

## Ethics Statement

This study was approved by the Ethics Committee on Animal Use on Research of the FMVZ/USP under protocol number 2361130722.

## Conflicts of Interest

The authors declare no conflicts of interest.

## Supporting information


**Figure S1:** Representative immunohistochemical photomicrographs of CD3+ and Iba1+ cells immunolabeling in the spleen and liver of howler monkeys (*Alouatta* spp.). (A) Membranous CD3+‐stained lymphocytes in the splenic white pulp of an uninfected animal, and (B) of a *Toxoplasma gondii*‐infected animal. (C) Cytoplasmic Iba1+‐stained cells in the liver periportal area of an uninfected animal, and (D) as part of a lymphohistiocytic infiltrate in a liver periportal necrotic area of an infected animal. DAB staining, 400×.

**Figure S2:** Representative immunohistochemical photomicrographs of CD3+ and Iba1+ cells immunolabeling in the spleen and liver of marmosets (*Callithrix* spp.). (A) Membranous CD3+‐stained lymphocytes in the splenic white pulp of an uninfected animal, and (B) of a *Toxoplasma gondii*‐infected animal. (C) Cytoplasmic Iba1+‐stained cells around a hepatic centrolobular vein (arrow indicates an Iba1+ Kupffer cell) of an uninfected animal, and (D) in a hepatic necrotic area of an infected animal. DAB staining, 400×.

**Figure S3:** Representative immunophotomicrographs of cytokine immunohistochemical (IHC) expression in splenic tissue of naturally *Toxoplasma gondii*‐infected howler monkeys (*Alouatta* sp.) and marmosets (*Callithrix* sp.). (A) Marked, multifocal IFN‐**γ** and (B) IL‐10 immunolabeling in splenic red pulp of howler monkeys. (C) IFN‐**γ** positive cells in the splenic red pulp and (D) IL‐10 immunolabeling in the splenic white pulp of marmosets. DAB, 400×.

**Table S1:** Epidemiological data of neotropical primates with confirmed toxoplasmosis.

**Table S2:** Epidemiological data of *Toxoplasma gondii*‐negative neotropical primates used as controls for splenic white pulp morphometry.

**Table S3:** Comparison between polymorphonuclear neutrophils (PMN), Iba1+ and CD3+ cell counts (mean ± SD—median), within each tissue, for howler monkeys (*Alouatta* spp.) and marmosets (*Callithrix* spp.).
**Table S4:** Comparison of polymorphonuclear neutrophils (PMN), Iba1+, and CD3+ cell counts (mean ± SD—median) between naturally *Toxoplasma gondii*‐infected howler monkeys (*Alouatta* spp.) and marmosets (*Callithrix* spp.) in liver, spleen, and central nervous system (CNS).

## Data Availability

The data that support the findings of this study are available within the article and its Supporting Information.
