# Supplementary material for: Comparative Immunopathology of Toxoplasma gondii Natural Infections in Marmosets (Callithrix spp.) and Howler Monkeys (Alouatta spp.), Brazil
Source: J Med Primatol. 2026 Sep 4;55(5):e70112. doi: 10.1111/jmp.70112 (PMC13545323; doi:10.1111/jmp.70112)
Supplement: Supplementary file 2 [file JMP-55-e70112-s002.pdf]

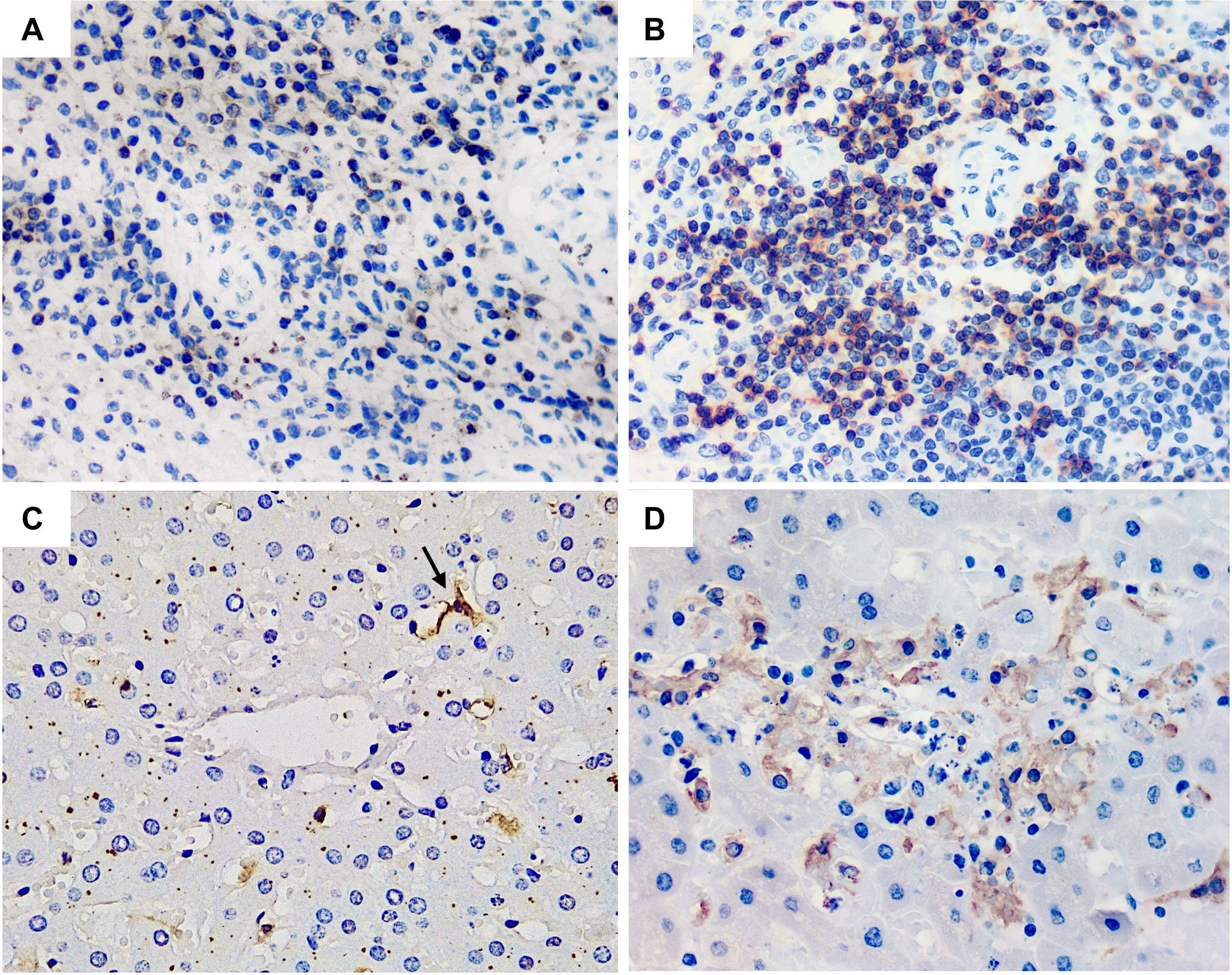

Figure S2. Representative immunohistochemical photomicrographs of CD3+ and Iba1+ cells immunolabeling in the spleen and liver of marmosets (*Callithrix* spp.). (A) Membranous CD3+-stained lymphocytes in the splenic white pulp of an uninfected animal, and (B) of a *Toxoplasma gondii*-infected animal. (C) Cytoplasmic Iba1+-stained cells around a hepatic centrolobular vein (arrow indicates an Iba1+ Kupffer cell) of an uninfected animal, and (D) in a hepatic necrotic area of an infected animal. DAB staining, 400×
