# Supplementary material for: Comparative Immunopathology of Toxoplasma gondii Natural Infections in Marmosets (Callithrix spp.) and Howler Monkeys (Alouatta spp.), Brazil
Source: J Med Primatol. 2026 Sep 4;55(5):e70112. doi: 10.1111/jmp.70112 (PMC13545323; doi:10.1111/jmp.70112)
Supplement: Supplementary file 3 [file JMP-55-e70112-s003.pdf]

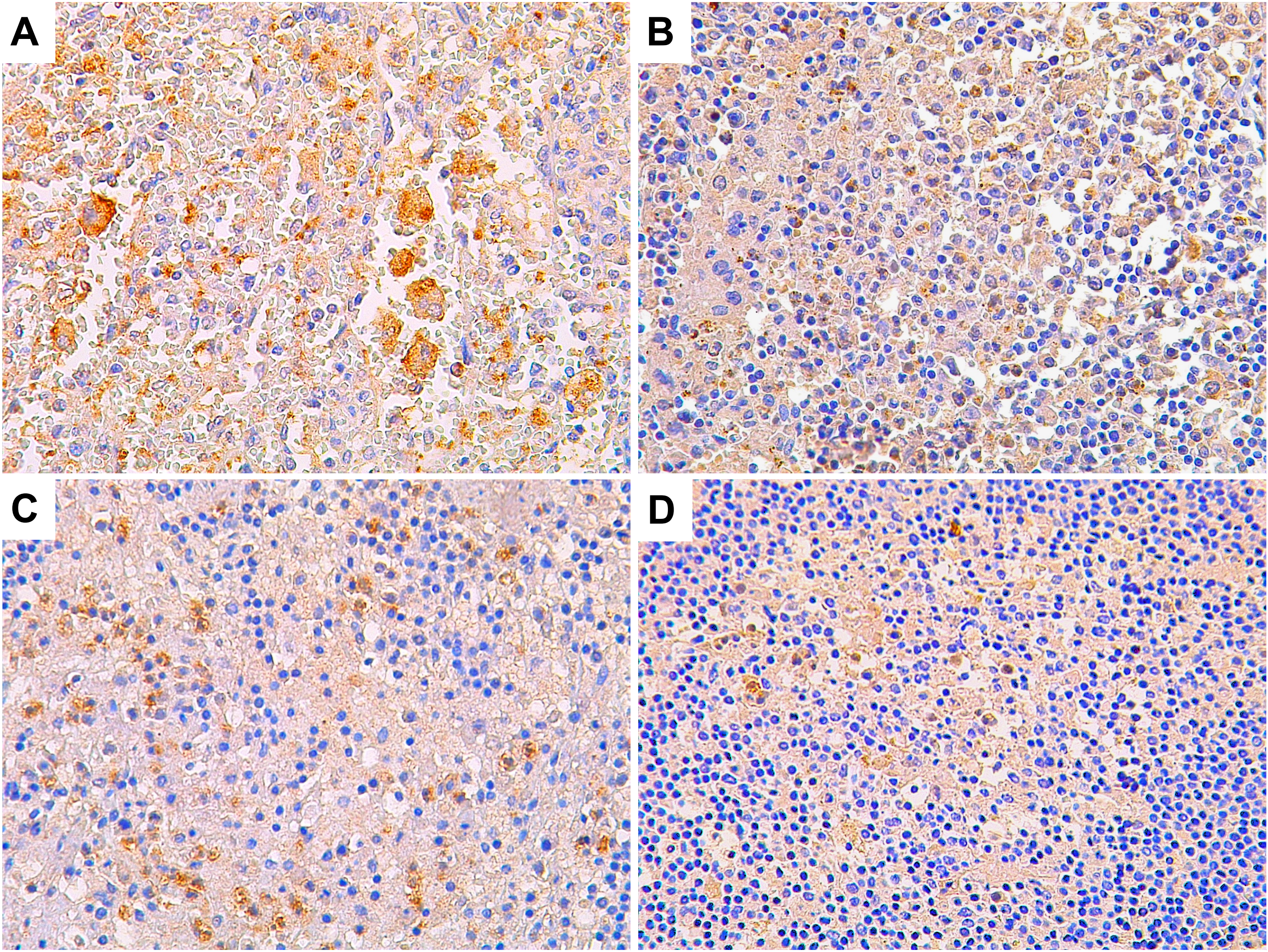

Figure S3. Representative immunophotomicrographs of cytokine immunohistochemical (IHC) expression in splenic tissue of naturally *Toxoplasma gondii*-infected howler monkeys (*Alouatta* sp.) and marmosets (*Callithrix* sp.). (A) Marked, multifocal IFN-ɣ and (B) IL-10 immunolabelling in splenic red pulp of howler monkeys. (C) IFN-ɣ positive cells in the splenic red pulp and (D) IL-10 immunolabelling in the splenic white pulp of marmosets. DAB, 400x
