# Supplementary material for: Comparative Immunopathology of Toxoplasma gondii Natural Infections in Marmosets (Callithrix spp.) and Howler Monkeys (Alouatta spp.), Brazil
Source: J Med Primatol. 2026 Sep 4;55(5):e70112. doi: 10.1111/jmp.70112 (PMC13545323; doi:10.1111/jmp.70112)
Supplement: Supplementary file 4 [file JMP-55-e70112-s004.docx]

**Supplementary Table S2.** Epidemiological data of Toxoplasma gondii-negative neotropical primates used as controls for splenic white pulp morphometry.

| ID | FAMILY | GENUS | SPECIES | SEX | AGE | Municipality | STATE | MONTH | YEAR | ORIGIN |
| --- | --- | --- | --- | --- | --- | --- | --- | --- | --- | --- |
| NHP-C1 | Callithrichidae | *Callithrix* | *Callithrix* sp. | Female | Adult | Brasília | DF | October | 2015 | NI |
| NHP-C2 | Callithrichidae | *Callithrix* | *Callithrix* sp. | Male | Adult | Brasília | DF | October | 2015 | NI |
| NHP-C3 | Atelidae | *Alouatta* | *Alouatta* sp. | Male | Subadult | Jundiái | SP | March | 2016 | NI |
| NHP-C4 | Callithrichidae | *Callithrix* | *Callithrix* sp. | Male | Subadult | São Paulo | SP | October | 2016 | NI |
| NHP-C5 | Callithrichidae | *Callithrix* | *Callithrix* sp. | NI | Subadult | Jundiái | SP | September | 2017 | NI |
| NHP-C6 | Callithrichidae | *Callithrix* | *Callithrix* sp. | NI | NI | Santa Clara d'Oeste | SP | Outubro | 2017 | NI |
| NHP-C7 | Callithrichidae | *Callithrix* | *Callithrix* sp. | NI | NI | São José do Rio Preto | SP | October | 2017 | NI |
| NHP-C8 | Callithrichidae | *Callithrix* | *Callithrix* sp. | Female | Adult | Jaguariúna | SP | October | 2017 | NI |
| NHP-C9 | Atelidae | *Alouatta* | *Alouatta* sp. | Male | Adult | São Paulo | SP | November | 2017 | NI |
| NHP-C10 | Callithrichidae | *Callithrix* | *Callithrix* sp. | Male | NI | Valinhos | SP | December | 2017 | NI |
| NHP-C11 | Atelidae | *Alouatta* | *Alouatta* sp. | Male | Adult | São Paulo | SP | December | 2017 | NI |
| NHP-C12 | Atelidae | *Alouatta* | *Alouatta* sp. | Male | Adult | São Paulo | SP | December | 2017 | NI |
| NHP-C13 | Callithrichidae | *Callithrix* | *Callithrix* sp. | Female | Adult | São José do Rio Preto | SP | February | 2017 | NI |
| NHP-C14 | Atelidae | *Alouatta* | *Alouatta* sp. | Female | Adult | São Paulo | SP | July | 2017 | NI |
| NHP-C15 | Atelidae | *Alouatta* | *Alouatta* sp. | Male | Adult | São Paulo | SP | December | 2017 | NI |
| NHP-C16 | Callithrichidae | *Callithrix* | *Callithrix* sp. | Female | NI | Taubaté | SP | August | 2018 | NI |
| NHP-C17 | Atelidae | *Alouatta* | *Alouatta* sp. | Male | Adult | São Paulo | SP | January | 2019 | NI |
| NHP-C18 | Callithrichidae | *Callithrix* | *Callithrix* sp. | Male | Subadult | Ribeirão Preto | SP | April | 2019 | NI |
| NHP-C19 | Atelidae | *Alouatta* | *Alouatta* sp. | Male | Subadulte | São Paulo | SP | May | 2019 | NI |
| NHP-C20 | Atelidae | *Alouatta* | *Alouatta* sp. | NI | NI | São Paulo | SP | May | 2019 | NI |
| NHP-C21 | Callithrichidae | *Callithrix* | *Callithrix* sp. | Male | Subadult | São Paulo | SP | June | 2019 | NI |
| NHP-C22 | Atelidae | *Alouatta* | *Alouatta* sp. | Male | Adult | Ibiúna | SP | July | 2019 | NI |
| NHP-C23 | Atelidae | *Alouatta* | *Alouatta* sp. | Male | Adult | Borborema | SP | September | 2019 | Free-living |
| NHP-C24 | Callithrichidae | *Callithrix* | *Callithrix* sp. | Female | Adult | Valinhos | SP | June | 2020 | NI |
| NHP-C25 | Callithrichidae | *Callithrix* | *Callithrix* sp. | Male | Subadult | São Paulo | SP | June | 2020 | NI |
| NHP-C26 | Callithrichidae | *Callithrix* | *Callithrix* sp. | Female | Adult | São Paulo | SP | September | 2020 | NI |
| NHP-C27 | Callithrichidae | *Callithrix* | *Callithrix* sp. | Male | Adult | Barueri | SP | March | 2021 | NI |
| NHP-C28 | Atelidae | *Alouatta* | *Alouatta* sp. | NI | NI | São Paulo | SP | March | 2021 | NI |
| NHP-C29 | Callithrichidae | *Callithrix* | *Callithrix* sp. | Male | Adult | São Paulo | SP | March | 2021 | NI |
| NHP-C30 | Callithrichidae | *Callithrix* | *Callithrix* sp. | Female | Subadult | São Paulo | SP | March | 2021 | NI |
| NHP-C31 | Callithrichidae | *Callithrix* | *Callithrix* sp. | Female | Adult | São Paulo | SP | March | 2021 | NI |
| NHP-C32 | Callithrichidae | *Callithrix* | *Callithrix* sp. | Male | Adult | Vinhedo | SP | April | 2021 | NI |
| NHP-C33 | Callithrichidae | *Callithrix* | *Callithrix* sp. | Female | Subadult | Ribeirão Preto | SP | June | 2021 | NI |
| NHP-C34 | Callithrichidae | *Callithrix* | *Callithrix* sp. | Male | Adult | São Paulo | SP | June | 2021 | NI |
| NHP-C35 | Callithrichidae | *Callithrix* | *Callithrix* sp. | NI | NI | Barueri | SP | July | 2021 | NI |
| NHP-C36 | Callithrichidae | *Callithrix* | *Callithrix* sp. | Male | Aged | Barueri | SP | August | 2021 | NI |
| NHP-C37 | Callithrichidae | *Callithrix* | *Callithrix* sp. | Female | Adult | São José dos Campos | SP | December | 2021 | Free-living |
| NHP-C38 | Atelidae | *Alouatta* | *Alouatta* sp. | Male | NI | São Paulo | SP | March | 2022 | NI |
| NHP-C39 | Callithrichidae | *Callithrix* | *Callithrix* sp. | Female | Adult | São Paulo | SP | July | 2022 | NI |
| NHP-C40 | Callithrichidae | *Callithrix* | *Callithrix* sp. | Female | Adult | São Paulo | SP | October | 2022 | NI |
| NHP-C41 | Callithrichidae | *Callithrix* | *Callithrix* sp. | Female | Adult | São José do Río Preto | SP | October | 2022 | Rural |
| NHP-C42 | Callithrichidae | *Callithrix* | *Callithrix* sp. | Female | Adult | São José do Río Preto | SP | November | 2022 | NI |
| NHP-C43 | Atelidae | *Alouatta* | *Alouatta* sp. | NI | NI | Itapecerica da Serra | SP | December | 2017 | NI |
