# Supplementary material for: Comparative Immunopathology of Toxoplasma gondii Natural Infections in Marmosets (Callithrix spp.) and Howler Monkeys (Alouatta spp.), Brazil
Source: J Med Primatol. 2026 Sep 4;55(5):e70112. doi: 10.1111/jmp.70112 (PMC13545323; doi:10.1111/jmp.70112)
Supplement: Supplementary file 5 [file JMP-55-e70112-s005.docx]

**Supplementary Table S1.** Epidemiological data of neotropical primates with confirmed toxoplasmosis.

| ID | Family | GENERA | Species | Sex | Age | Municipality | State | Month | Year | ORIGIN |
| --- | --- | --- | --- | --- | --- | --- | --- | --- | --- | --- |
| NHP-001 | Callitrichidae | *Callithrix* | *Callithrix penicillata* | Male | Adult | Brasília | DF | October | 2015 | NI |
| NHP-002 | Callitrichidae | *Callithrix* | *Callithrix penicillata* | Female | Adult | Brasília | DF | October | 2015 | NI |
| NHP-003 | Callitrichidae | *Callithrix* | *Callithrix penicillata* | Female | Subadult | Brasília | DF | October | 2015 | NI |
| NHP-004 | Callitrichidae | *Callithrix* | *Callithrix penicillata* | Female | Adult | Brasília | DF | October | 2015 | NI |
| NHP-005 | Callitrichidae | *Callithrix* | *Callithrix penicillata* | Female | Subadult | Brasília | DF | October | 2015 | NI |
| NHP-006 | Callitrichidae | *Callithrix* | *Callithrix jacchus* | NI | NI | Olímpia | SP | November | 2015 | NI |
| NHP-007 | Callitrichidae | *Callithrix* | *Callithrix jacchus* | NI | NI | Olímpia | SP | November | 2015 | NI |
| NHP-008 | Callitrichidae | *Callithrix* | *Callithrix penicillata* | NI | NI | Americana | SP | November | 2016 | Captivity |
| NHP-009 | Callitrichidae | *Callithrix* | *Callithrix penicillata* | Female | Aged | Ribeirão Preto | SP | March | 2017 | Urban |
| NHP-010 | Callitrichidae | *Callithrix* | *Callithrix* sp. | NI | NI | Palmeira do Este | SP | April | 2017 | Urban |
| NHP-011 | Callitrichidae | *Callithrix* | *Callithrix* sp. | Male | Adult | Altair | SP | April | 2017 | Urban |
| NHP-012 | Callitrichidae | *Callithrix* | *Callithrix* sp. | NI | NI | Pedranópolis | SP | May | 2017 | Rural |
| NHP-013 | Callitrichidae | *Callithrix* | *Callithrix jacchus* | NI | NI | Umbauba | SE | June | 2017 | NI |
| NHP-014 | Callitrichidae | *Callithrix* | *Callithrix* sp. | Female | NI | São José do Rio Preto | SP | June | 2017 | Urban |
| NHP-015 | Callitrichidae | *Callithrix* | *Callithrix* sp. | NI | NI | Macedonia | SP | March | 2018 | Rural |
| NHP-016 | Callitrichidae | *Callithrix* | *Callithrix penicillata* | NI | NI | Campinas | SP | May | 2018 | Urban |
| NHP-017 | Callitrichidae | *Callithrix* | *Callithrix* sp. | Female | Adult | São José do Rio Preto | SP | July | 2018 | Urban |
| NHP-018 | Callitrichidae | *Callithrix* | *Callithrix* sp. | Male | Adult | São José do Rio Preto | SP | July | 2018 | Urban |
| NHP-019 | Callitrichidae | *Callithrix* | *Callithrix penicillata* | Male | Aged | Ribeirão Preto | SP | July | 2018 | Urban |
| NHP-020 | Callitrichidae | *Callithrix* | *Callithrix* sp. | Male | Adult | Jandira | SP | August | 2018 | Urban |
| NHP-021 | Callitrichidae | *Callithrix* | *Callithrix* sp. | Female | Subadult | Ribeirão Preto | SP | September | 2018 | Urban |
| NHP-022 | Callitrichidae | *Callithrix* | *Callithrix* sp. | NI | NI | Barueri | SP | October | 2018 | NI |
| NHP-023 | Callitrichidae | *Callithrix* | *Callithrix penicillata* | Male | Adult | Ribeirão Preto | SP | December | 2018 | Urban |
| NHP-024 | Callitrichidae | *Callithrix* | *Callithrix* sp. | Male | NI | São José do Rio Preto | SP | February | 2019 | Urban |
| NHP-025 | Callitrichidae | *Callithrix* | *Callithrix penicillata* | NI | NI | Americana | SP | March | 2019 | Captivity |
| NHP-026 | Callitrichidae | *Callithrix* | *Callithrix penicillata* | Male | Adult | Ribeirão Preto | SP | May | 2019 | Urban |
| NHP-027 | Callitrichidae | *Callithrix* | *Callithrix penicillata* | Female | Adult | Ribeirão Preto | SP | November | 2019 | Urban |
| NHP-028 | Callitrichidae | *Callithrix* | *Callithrix* sp. | NI | NI | Mongaguá | SP | June | 2020 | NI |
| NHP-029 | Callitrichidae | *Callithrix* | *Callithrix* sp. | Male | Adult | Valinhos | SP | June | 2020 | Urban |
| NHP-030 | Callitrichidae | *Callithrix* | *Callithrix jacchus* | NI | NI | Olímpia | SP | November | 2015 | NI |
| NHP-031 | Callitrichidae | *Callithrix* | *Callithrix* sp. | Male | Adult | São José do Rio Preto | SP | November | 2016 | Urban |
| NHP-032 | Atelidae | *Alouatta* | *Alouatta guariba clamitans* | Male | NI | Indaial | SC | May | 2015 | NI |
| NHP-033 | Atelidae | *Alouatta* | *Alouatta guariba clamitans* | Female | Adult | Blumenau | SC | May | 2015 | NI |
| NHP-034 | Atelidae | *Alouatta* | *Alouatta* sp. | Macho | Subadult | Blumenau | SC | August | 2015 | NI |
| NHP-035 | Atelidae | *Alouatta* | *Alouatta* sp. | NI | NI | Olímpia | SP | November | 2015 | NI |
| NHP-036 | Atelidae | *Alouatta* | *Alouatta guariba clamitans* | Male | Adult | Florianópolis | SC | June | 2016 | NI |
| NHP-037 | Atelidae | *Alouatta* | *Alouatta guariba clamitans* | Male | Adult | Blumenau | SC | August | 2016 | NI |
| NHP-038 | Atelidae | *Alouatta* | *Alouatta guariba clamitans* | Female | Juvenile | Indaial | SC | December | 2016 | Captivity |
| NHP-039 | Atelidae | *Alouatta* | *Alouatta guariba clamitans* | NI | Adult | São Sepe | RS | February | 2017 | Forest |
| NHP-040 | Atelidae | *Alouatta* | *Alouatta* sp. | NI | NI | Jundiaí | SP | March | 2017 | NI |
| NHP-041 | Atelidae | *Alouatta* | *Alouatta guariba clamitans* | Male | Adult | Blumenau | SC | May | 2017 | Forest |
| NHP-042 | Atelidae | *Alouatta* | *Alouatta* sp. | Male | Adult | São Bento do Sul | SC | June | 2017 | Urban |
| NHP-043 | Atelidae | *Alouatta* | *Alouatta guariba* | Male | Adult | Jundiaí | SP | June | 2017 | Rural |
| NHP-044 | Atelidae | *Alouatta* | *Alouatta guariba* | Female | Adult | Sapé | PB | June | 2017 | Rural |
| NHP-045 | Atelidae | *Alouatta* | *Alouatta guariba clamitans* | NI | NI | Igrejinha | RS | June | 2017 | Rural |
| NHP-046 | Atelidae | *Alouatta* | *Alouatta guariba clamitans* | Female | Adult | Blumenau | SC | August | 2017 | Urban |
| NHP-047 | Atelidae | *Alouatta* | *Alouatta guariba clamitans* | Male | Adult | São Paulo | SP | April | 2018 | Urban |
| NHP-048 | Atelidae | *Alouatta* | *Alouatta guariba clamitans* | Macho | Adult | São Paulo | SP | May | 2019 | Urban |
| NHP-049 | Atelidae | *Alouatta* | *Alouatta guariba clamitans* | Femea | Adult | São Paulo | SP | July | 2019 | Captivity |
| NHP-050 | Atelidae | *Alouatta* | *Alouatta guariba clamitans* | Femea | Aged | Guarulhos | SP | July | 2018 | Captivity |
