# Supplementary material for: Comparative Immunopathology of Toxoplasma gondii Natural Infections in Marmosets (Callithrix spp.) and Howler Monkeys (Alouatta spp.), Brazil
Source: J Med Primatol. 2026 Sep 4;55(5):e70112. doi: 10.1111/jmp.70112 (PMC13545323; doi:10.1111/jmp.70112)
Supplement: Supplementary file 6 [file JMP-55-e70112-s006.docx]

Supplementary Table S3. Comparison between polymorphonuclear neutrophils (PMN), Iba1+ and CD3+ cell counts (mean ± SD – median), within each tissue, for howler monkeys (Alouatta spp.) and marmosets (Callithrix spp.)

| Group | Tissue | PMN | Iba1+ | CD3+ | *p*-value |
| --- | --- | --- | --- | --- | --- |
| **Howler monkeys** | Liver | 9.19 ± 3.60 – 9.40 | 43.4 ± 25.5 – 42.5 | 8.83 ± 11.74 – 2.67 | <0.001^1^ |
|  | Spleen | 13.4 ± 5.4 – 12.9 | 53.8 ± 23.3 – 57.0 | 51.3 ± 22.8 – 52.5 | <0.001^1^ |
|  | CNS | 4.76 ± 4.49 – 2.10 | 46.9 ± 14.5 – 48.7 | 3.38 ± 3.13 – 4.10 | 0.009^1^ |
| **Marmosets** | Liver | 11.4 ± 5.8 –b 11.9 | 34.1 ± 19.0 – 29.7 | 8.61 ± 9.95 – 4.30 | <0.001^1^ |
|  | Spleen | 16.8 ± 8.4 – 14.4 | 73.2 ± 26.6 – 68.5 | 103.4 ± 66.2 – 100.8 | <0.001^1^ |
|  | CNS | 3.84 ± 2.02 – 3.40 | 27.1 ± 23.7 – 16.5 | 0.79 ± 0.74 – 0.70 | <0.001^1^ |
| Cell counts expressed as mean ± SD - median. p-value from Kruskal-Wallis test comparing the three cell types within each tissue. ^1^ *p* < 0.05. SD, standard deviation; CNS, central nervous system; PMN, polymorphonuclear neutrophil. | | | | | |

Supplementary Table S4. Comparison of polymorphonuclear neutrophils (PMN), Iba1+, and CD3+ cell counts (mean ± SD – median) between naturally Toxoplasma gondii-infected howler monkeys (Alouatta spp.) and marmosets (Callithrix spp.) in liver, spleen, and central nervous system (CNS).

| Cell type | Tissue | Howler monkeys, (n = 18) | Marmosets, (n = 29) | *p*-value |
| --- | --- | --- | --- | --- |
| **PMN** | Liver | 9.19 ± 3.60 -- 9.40 | 11.4 ± 5.8 – 11.9 | 0.304 |
|  | Spleen | 13.4 ± 5.4 –12.9 | 16.8 ± 8.4 – 14.4 | 0.294 |
|  | CNS | 4.76 ± 4.49 – 2.10 | 3.84 ± 2.02 – 3.40 | 0.687 |
| **Iba1+** | Liver | 43.4 ± 25.5 – 42.5 | 34.1 ± 19.0 – 29.7 | 0.232 |
|  | Spleen | 53.8 ± 23.3 – 57.0 | 73.2 ± 26.6 – 68.5 | 0.030^1^ |
|  | CNS | 46.9 ± 14.5 – 48.7 | 27.1 ± 23.7 – 16.5 | 0.104 |
| **CD3+** | Liver | 8.83 ± 11.74 – 2.67 | 8.61 ± 9.95 – 4.30 | 0.839 |
|  | Spleen | 51.3 ± 22.8 – 52.5 | 103.4 ± 66.2 – 100.8 | 0.002^1^ |
|  | CNS | 3.38 ± 3.13 – 4.10 | 0.79 ± 0.74 – 0.70 | 0.138 |
| Cell counts expressed as mean ± SD - median. p-value from Student's t-test (Welch) or Mann-Whitney U test, chosen per comparison based on the Shapiro-Wilk normality test. ^1^*p* < 0.05 (Student's t-test). SD, standard deviation; CNS, central nervous system; PMN, polymorphonuclear neutrophils | | | | |
